# S195. ELECTRORETINOGRAPHIC INDICES OF PHOTORECEPTOR, BIPOLAR, AND GANGLION CELL FUNCTIONING DIFFERENTIATE PEOPLE WITH SCHIZOPHRENIA FROM THOSE WITH MAJOR DEPRESSION AND HEALTHY CONTROLS

**DOI:** 10.1093/schbul/sby018.982

**Published:** 2018-04-01

**Authors:** Docia Demmin, Matthew Roché, Roni Netser, Steven Silverstein

**Affiliations:** 1 Rutgers University; 2 New Jersey City University

## Abstract

**Background:**

The retina is part of the CNS and provides a window into brain structure and function that has been useful in examining schizophrenia and other psychiatric disorders.

**Methods:**

In this ongoing study, we are using flash electroretinography (fERG) to compare retinal cell functioning in schizophrenia (n = 25) and major depressive disorder (MDD; n = 18, to date), both relative to psychiatrically healthy controls (n = 25). Data were averaged over both eyes and collected under both light- and dark-adapted conditions. The primary variables of interest were a-wave activity (reflecting photoreceptor response), b-wave activity (reflecting primarily bipolar cell activity), and photopic negative response (PhNR; reflecting ganglion cell activity).

**Results:**

On light-adapted (photopic) tests, schizophrenia patients demonstrated significantly weaker cone and bipolar cell responses than the MDD and healthy control groups (ds = .76 to 1.25). On dark-adapted (scotopic) tests, all groups demonstrated a linear increase in photoreceptor and bipolar cell response with increases in stimulus intensity, but the rate of response gain per unit of intensity increase was significantly weaker for schizophrenia patients than for the other groups (ds = .84 to 1.11). Significant group differences were also found in PhNR amplitude, with the schizophrenia group demonstrating a weaker PhNR (measured at 72 ms post-stimulus presentation) as compared to the healthy control group (d = .50). In the MDD group, the minimum PhNR amplitude occurred significantly earlier than in either of the other two groups (ds =.80 to .92).

**Discussion:**

These data confirm abnormal retinal cell functioning in schizophrenia patients receiving treatment. Our finding of normal retinal waveform amplitudes in MDD is consistent with a prior report of normalized fERG amplitudes after antidepressant treatment (Fornaro et al., 2011, J Affective Disorders), but inconsistent with another report showing abnormal values in treated MDD patients (Hébert et al., 2017, Prog Neuropsychopharmacol Biol Psychiatry). Our finding of enhanced PhNR implicit time in MDD is consistent, however, with evidence of enhanced amplitudes in untreated MDD patients in Fornaro et al. (2011). Further studies, using a range of fERG parameter values, are necessary to determine trait and state effects on retinal function in MDD, and which of these effects may overlap or contrast with what is observed in schizophrenia.

